# The clonal root system of balsam poplar in upland sites of Quebec and Alberta

**DOI:** 10.1002/ece3.2441

**Published:** 2016-09-06

**Authors:** Kokouvi E. Adonsou, Annie DesRochers, Francine Tremblay, Barb R. Thomas, Nathalie Isabel

**Affiliations:** ^1^ Institut de recherche sur les forêts Université du Québec en Abitibi‐Témiscamingue Amos QC Canada; ^2^ Institut de recherche sur les forêts Université du Québec en Abitibi‐Témiscamingue Rouyn‐Noranda QC Canada; ^3^ Department of Renewable Resources University of Alberta Edmonton AB Canada; ^4^ Natural Resources Canada Canadian Forest Service Laurentian Forestry Centre Québec QC Canada

**Keywords:** asexual reproduction, balsam poplar, clonal diversity, communal root system, root connection root suckering, single‐nucleotide polymorphism

## Abstract

Balsam poplar seeds are short‐lived and require moist seedbeds soon after they are released to germinate. In addition to sexual reproduction, balsam poplar stands can regenerate clonally by root suckering. The origin of stands will in turn affect their genetic structure and root system architecture, which are poorly understood for upland forest stands. Three stands were hydraulically excavated in Quebec (moist) and Alberta (dry) to determine the origin of trees and to characterize root systems with respect to presence of parental roots and root grafts connections. Clones were identified using single‐nucleotide polymorphism (SNPs), and all stems, roots and root grafts were aged using dendrochronology techniques. All 82 excavated trees were of sucker origin, and four of the six stands contained a single clone. Parental root connections were found between 22% and 25% of excavated trees, and 53% and 48% of trees were linked with a root graft between the same or different clones, in Alberta and Quebec, respectively. Mean distance between trees connected by parental root was significantly lower than the distance between unconnected trees (0.47 ± 0.25 m vs. 3.14 ± 0.15 m and 1.55 ± 0.27 m vs. 4.25 ± 0.13 m) in Alberta and in Quebec, respectively. The excavations also revealed many dead stumps with live roots, maintained through root connections with live trees. This research highlights that balsam poplar growing in upland stands is a clonal species that can maintain relatively high genotypic diversity, with frequent root connections between trees at maturity. Maintaining an extensive root system through root connections increases the chances of a clone surviving when the above ground tree is dead and may also enhance the resilience of balsam poplar stands after disturbance.

## 1 Introduction

Clonal growth directly affects the distribution of phenotypic and genetic variation within and among populations (Eckert, 1999). Knowledge of regeneration dynamics of a clonal species is thus essential to understand community structure and ecosystem functioning (Wilsey, 2002). Although recruitment from seed is important for many species and may be favored under certain environmental conditions (Kanno & Seiwa, 2004), many forest plants are capable of asexual propagation. Balsam poplar (*Populus balsamifera* L.) is distributed throughout North America and is mostly found in riparian mixed wood forests and on moister upland sites (Rood, Hillman, Sanche, & Mahoney, 1994). In addition to sexual reproduction, balsam poplar also propagates clonally, the extent of which is poorly understood (Gom & Rood, 1999). Vegetative regeneration can occur via root suckering, adventitious shoots emerging from shallow horizontal roots from established, cut or burned trees, and from buried excised branch fragments (Shaw, 1991; Wijesinghe & Hutchings, 1997; Zasada et al., 1981). However, its regeneration dynamics, including root system architecture, size of parental roots at the time of suckering as well as root grafting occurrence, are not documented for upland sites within the boreal forest. Root suckering is generally thought to be less important in balsam poplar following natural disturbance or harvesting when compared to the abundant root suckering observed in aspen (*Populus tremuloides* Michx.; Peterson & Peterson, 1992) as reported by Kemperman and Barnes (1976). Several studies along rivers in Alberta (Canada) have shown that saplings in riparian habitat originate more commonly from seedling establishment, whereas those located further away from the river edge are often of sucker origin (Kalischuk, Rood, & Mahoney, 2001; Rood et al., 1994). Colonization by root suckers is thus believed to be more important on dry sites where the probability of seedling recruitment is low (Krasny, Vogt, & Zasada, 1988). Suckering can lead to the formation of extensive clones (Edwards & Dunwiddie, 1985) in which suckers may be interconnected by parental roots (DesRochers & Lieffers, 2001b). Such diverse mechanisms of regeneration (sexual and asexual) of the species after disturbance or clear cutting potentially have strong effects on the genetic structure and diversity of the species. This genetic diversity appears necessary to ensure resilience and adaptation of balsam poplar under current and projected climate change (Eriksson, Namkoong, & Roberts, 1996).

The extent of root suckering with respect to sexual reproduction in balsam poplar forest settings (nonriparian) is currently unknown. Moreover, the occurrence of root grafts or the persistence of parental root connections between trees is unknown in balsam poplar. It has been shown in aspen that the original parental root connections remain alive throughout the life of the ramets and that the level of interconnection between trees can even increase with the formation of many root grafts (DesRochers & Lieffers, 2001b). More recently, Jelínková, Tremblay, and DesRochers (2009) showed how natural root grafting in aspen produced physiologically integrated clones, with many root grafts between trees of the same or different clones. Seed regeneration should normally increase genetic diversity of populations (Soane & Watkinson, 1979), which in turn is influenced by seed characteristics and dispersal capacity. Balsam poplar seeds are characterized by wide‐range dispersion, short‐term viability, moisture and mineral soil exposure dependence for germination, and early survival (Hosie, 1990; Krasny et al., 1988; Moss, 1938). Seed regeneration may therefore be related to precipitation patterns, the more abundant rain favoring seed regeneration over sucker regeneration. Regeneration mechanisms are thus possibly different between western (Alberta) and eastern (Quebec) Canada, with approximately double the amount of precipitations in the east (889 mm), where seedling recruitment would be expected to be greater than in the west (453 mm). An improved understanding of the regeneration mechanisms of the species can help sustainably manage forests, by contributing to selection strategies of parent material for tree improvement programs or guiding government policy on clonal deployment on the landscape in order to mimic natural scenarios.

The goals of this study were to: (1) investigate natural regeneration patterns, by root suckering versus seedlings of balsam poplar; (2) characterize the architecture of the root system (presence or absence of root connections between trees) in naturally established balsam poplar stands on upland sites; and (3) compare results obtained for each of both regions that have similar climates but contrasting precipitation levels. Because balsam poplar seeds do not exhibit dormancy and germination occurs immediately on moist mineral sites (Zasada & Densmore, 1980), we hypothesized that (1) there would be more seedling recruitment in Quebec than in semiarid Alberta and (2) that similarly as for aspen, most ramets of a given clone that have regenerated from the same parental root system would remain interconnected through mature stages of stand development.

## 2 Materials and Methods

### 2.1 Study areas

Six balsam poplar stands on mesic upland sites were selected in two provinces (regions) of Canada (three in Quebec and three in Alberta). In each region, typical monospecific balsam poplar stands (>75% of stems) of natural origin established after stand‐replacing fires were selected adjacent to a gentle slope with a water supply nearby for hydraulic excavation of root systems. All sites within a region were at least 1 km away from each other.

Two of the Quebec sampling sites (QC1:48°29′N, 79°24′W, 287 m a.s.l. and QC2: 48°24′N, 79°24′W 304 m a.s.l.) were located near the Lake Duparquet Research and Teaching Forest (48°28′N, 79°17′W), and a third site (QC3: 48°32′N, 78°08′W, 305 m a.s.l.) was located near Amos (48°36′N, 78°04′W) in the Abitibi region of northwestern Quebec. This region is part of the Northern Clay Belt of Quebec and Ontario, which resulted from the maximum extension of the postglacial Lakes Barlow and Ojibway (Vincent & Hardy, 1977). The nearest meteorological station to two study sites (QC1, QC2) is at La Sarre, approximately 42 km to the north, while that of the third site (QC3) is at Val d'Or around 67 km to the south. The average climate for the three decades 1971–2000 showed a mean annual temperature of 0.7°C and mean total annual precipitation of 889 mm, with 643 mm as rainfall during the growing season (Environment Canada 2012). The mean frost‐free period is 64 days, but freezing can occur at any time of the year (Environment Canada 2012). The average growing season with temperatures ranges from 13.2 to 15.9°C and varies between 153 and 177 days. In the lower areas, lacustrine clay soils derived from the postglacial Lakes Barlow and Ojibway are the primary surface deposits; tills and sandy fluvio‐glacial soils dominate the higher ground (Vincent & Hardy, 1977). This study region is located at the southern limit of the boreal forest, where most stands originate from natural forest fires and are dominated by an association of aspen and balsam poplar, *Abies balsamea* (L) Mill. *Picea mariana* (Mill.) BSP, *Betula papyrifera* (Marsh), and *Picea glauca* (Moench) Voss.

The three sampling sites in Alberta were located near the town of Athabasca (54°49′N, 113°32′W), 152 km north of the city of Edmonton. Sites AB1 (55°11′N, 113°01′W, 590 m a.s.l.), AB2 (55°11′N, 113°02′W, 606 m a.s.l.), and AB3 (55°13′N, 113°03′W, 616 m a.s.l.) were selected based on the same criteria as for the Quebec sites. This region belongs to the central dry mixed wood subregion of Alberta (Beckingham & Archibald, 1996). The closest meteorological station of this study area is at Calling Lake approximately 15 km to the north. Mean annual temperature is 0.9°C and mean annual precipitation is 453 mm, with an average of 349 mm occurring as rainfall during the growing season (Environment Canada 2012). The average growing season with temperatures >10°C is approximately 170 days. Typical mineral soils in the areas are dark brown or black Chernozems developed under mixed deciduous vegetation. The three study sites had similar vegetation and were characterized as aspen and balsam poplar codominated on moderately well‐drained mesic sites (Beckingham & Archibald, 1996).

### 2.2 Sampling

**Figure 1:**
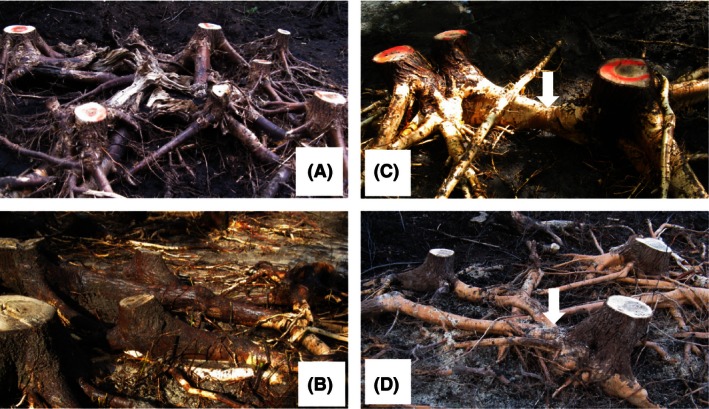
Photographs of excavated root systems of balsam poplar showing main root system at site AB1 (A) in Alberta and site QC1 (B) in Quebec and clonal integration: the arrows indicate parental root connection between trees at site AB2 (C) in Alberta and interclonal root graft between trees at site QC3 (D) in Quebec, Canada

Similar to Tarroux and DesRochers (2011), we used a high‐pressure water spray from a forest fire pump (Mark III, WAJAX Lachine, Quebec) to excavate the root systems. Each excavated plot was 50–60 m^2^ and included at least 10 trees (Table 1). We measured tree diameter at breastheight (DBH: 1.3 m) and distance between trees before felling all trees and measuring their heights. Mean soil organic matter thickness was 10.4 cm in Quebec and 26.7 cm in Alberta, which was measured on a diagonal for each plot (one in the center and two in opposite corners) and averaged. Depth of excavation to expose all main roots varied from 30 to 60 cm (Fig. 1A,B). Following excavation, we mapped all trees, stumps, roots, and root connections between trees. For growth‐ring analysis, we collected cross‐sectional disks of all stems at breastheight and at ground level (0 m), root connections and root grafts between trees and cross‐sectional disks from each root (diameter >2 cm), parental and nonparental roots (hereafter named “new roots” because they were produced after stand establishment and are thus younger than the trees on which they are growing) where they connect to the stump. Roots are larger closer to the stump and have fewer missing rings, and annual growth rings are easier to observe (Krause & Morin, 1999). In total, we sampled 43 trees in Quebec and 39 trees in Alberta.

**Table 1:** Characteristics of the six excavated sites of balsam poplar stand in Alberta and in Quebec (standard errors of the mean are given in parentheses)

Sites	Alberta			Quebec		
	AB1	AB2	AB3	QC1	QC2	QC3
Number of excavated trees	14	15	10	11	16	16
Age of trees	43	43	103	86	87	44
Mean DBH (cm)	16.31 (7.36)	15.90 (5.42)	32.52 (7.04)	33.69 (6.53)	29.61 (6.62)	20.91 (6.42)
Mean height (m)	16.03 (2.52)	16.54 (0.30)	19.78 (3.49)	23.28 (2.74)	20.86 (2.57)	18.46 (2.16)
Size of excavated area (m^2^)	50	50	60	50	50	50
Soil organic matter (cm)	27.85 (0.56)	25.5 (0.91)	26.65 (1.02)	10.26 (0.25)	10.31 (0.16)	10.61 (0.12)

### 2.3 Dendrochronological analysis and root measurements

After 3 months air‐drying, all stem and root cross‐sectional disks were progressively sanded (80–400 grit paper) to clearly reveal growth rings. We counted growth rings using a binocular microscope. The distinction between root growth rings was more difficult than in stems because of eccentricity of cross sections and the existence of frequent discontinuous and narrow rings (Fayle, 1975; Krause & Eckstein, 1994; Tarroux & DesRochers, 2011). To improve visibility of small growth rings or to obtain a better ring‐to‐ring contrast, the surface of root cross sections was cut with a sharp razor blade and wood cells filled with white chalk (DesRochers & Lieffers, 2001b; Tarroux & DesRochers, 2011). To accurately determine the age of each individual tree, each ground level cross section was cross‐dated with their corresponding breastheight cross section using pointer years, for example, narrow or wide rings (Schweingruber, 1988). The age of each root cross section was then compared with the age of its corresponding tree in order to distinguish the origin of each tree, root sucker versus seedling. If we found roots older than its corresponding tree, those were labeled as parental roots and trees were determined to be from a sucker origin. If all roots were younger than their corresponding tree, we considered those trees to have established from seedling recruitment.

Root grafts were air‐dried for 3 months and then cut into cross‐sectional disks throughout the length of the graft in order to confirm whether or not the grafted roots shared common growth rings (true morphological union). Age of grafts was determined by counting the number of continuous growth rings shared by the grafted roots.

Root diameter (parental and new roots) was measured using a technique similar to that described by Ruel, Larouche, and Achim (2003) and Di Iorio, Lasserre, Scippa, and Chiatante (2005). Roots were assumed to be either circular in cross‐sectional disks or elliptical with large and short diameters, and diameter of the root disk was calculated as the mean of perpendicular measurements of the longest and the shortest diameters of the root. Root cross‐sectional area was calculated as the area of a circle using the corresponding mean diameter. Thus, the ratio size parental root to size new root (P/N) was calculated by dividing the area of the parental roots by the area of new roots. The ratio of parental to new roots between the two regions (Quebec or Alberta) was tested to assess the importance of the parental root system using root area (size) as a proxy for biomass. To characterize the roots from which our sampled trees originated, diameter of the parental roots at the time of suckering was determined by measuring its diameter before suckering occurred, that is, by subtracting from its current size the growth rings that were produced after suckering.

Root diameter and distance between trees (connected or not) were tested to better characterize the architecture of the root systems and to verify whether trees growing closer to one another had better chances of remaining connected by parental roots or by forming root grafts.

### 2.4 Clone identification

To discriminate intraclonal from interclonal root grafting occurrence, leaf samples and cambial tissue from the bark were collected from all trees and living roots from dead stumps. All samples were dried in silica gel and stored at −80°C. We extracted DNA from dried tissue using the Nucleospin 96 Plant II kit (Macherey‐Nagel, Bethlehem, Pennsylvania) and a vacuum manifold according to the manufacturer's protocols with the following modifications: (1) cell lysis using buffers PL2 and PL3 for 2 hr at 65°C and (2) elution with an in‐house Tris–HCl 0.01 mol/L pH 8.0 buffer. Then, DNA samples were genotyped with an assay of 36 well‐characterized SNPs of balsam poplar (average minor allele frequency of 0.26 has been estimated for more than 1,000 trees distributed over the entire natural range of the species) (Isabel et al., *submitted*). The SNPs were genotyped with the Sequenom iPlex Gold technology (Agena Bioscience, San Diego, California) at the McGill University, Génome Québec Innovation Centre using their internal protocols. The variability at these loci was sufficient to assign the 82 trees and 12 dead stumps to clones with high confidence. Pairwise comparisons of the genetic profiles obtained for each individual were manually conducted in order to detect any variations. When no genetic variation was observed among trees from the same stand, they were delineated as a clone.

### 2.5 Statistical analyses

Mixed models linear analyses of variance were performed to investigate size of parent root at the time of suckering, root growth (parental roots and new roots), ratio size of parental roots to size of new roots (P/N), and distance between trees connected by parental root or by root grafts. All data were tested setting province (Alberta and Quebec) as main factor and sites nested as random variables within province, using JMP statistical software (Version 11.2; SAS Institute Inc., Cary, NC, USA). For size of parental roots and root growth analyses, age of trees was used as covariate in order to compensate for age differences between trees across all sites. All data were checked for deviations from normality, and for homoscedasticity and logarithm, transformations were performed for the diameter of parental roots at the time of suckering, distance between trees connected by parental roots or by root grafts, and for ratio (P/N). ANOVAs were followed with post hoc comparisons, when significant effects occurred and choosing a significance level of *p* < .05.

Differences in the frequencies of grafted trees from nongrafted and trees connected or not by parental roots were statistically tested using χ^2^ analyses.

## 3 Results

### 3.1 Dendrochronological analysis and tree origin

All excavated trees were of sucker origin, since roots older than the stems (parental roots, Fig. 1C) were found in the root system of all trees. The age of sampled trees was 86, 87, 44 and 43, 43, 103 in Quebec and Alberta, respectively, and was even‐aged, that is, age of trees corresponded to time since fire. The maximum age difference found between parental roots and tree age was 51 and 11 years in Alberta and Quebec, respectively.

At the time of excavation, ratio of the size of parental to new roots (P/N) was significantly greater (*F*_1,3.3_ = 27.8, *p *<* *.001, Table 2) in Alberta than in Quebec (1.15 ± 0.06 vs. 0.70 ± 0.06, Table 3). Mean root diameter of parental roots at the time of suckering was also significantly greater (*F*_1,4.5_ = 49.2, *p *<* *.001, Table 2) in Alberta than in Quebec (1.2 vs. 0.83 cm, respectively Table 3). There was also a significant difference (*F*_1,1.6_ = 54.87 *p *=* *.02, Table 2) in diameter of new roots, with larger roots found in Quebec than in Alberta (17.47 ± 0.4 vs. 12.81 ± 0.4, Table 3).

**Table 2:** Analyses of variance showing sources of variation, degrees of freedom (DF, DFD), and *F* and *Pr* values for diameter of parental root, diameter of new root, ratio size of parental root/size of new root (P/N)

Source of variation	DF	DFDen	*F* value	*Pr* values
Diameter of parental root at the time of suckering				
Age of tree covariate	1	10.98	19.97	**0.001**
Provinces (Alberta/Quebec)	1	4.50	49.27	**0.001**
Diameter of new root at the time of excavation				
Age of tree covariate	1	2.51	8.50	0.076
Provinces (Alberta/Quebec)	1	1.65	54.87	**0.029**
Ratio size of parental root/size of new root (P/N)				
Provinces (Alberta/Quebec)	1	3.38	27.80	**0.009**

Bold indicates significant effects.

**Table 3:** Mean diameter (cm) of parental and new roots in the excavated balsam poplar stands in Alberta and Quebec (standard errors of the mean are given in parentheses)

Sites	Alberta				Quebec			
	AB1	AB2	AB3	Mean	QC1	QC2	QC3	Mean
Parental roots at the time of suckering (cm)	1.39 (0.53)	1.28 (0.39)	1.02 (0.26)	1.20 (0.03)	0.67 (0.44)	0.7 (0.43)	0.96 (0.55)	0.83 (0.03)
New roots at the time of excavation (cm)	12.85 (0.39)	12.77 (0.39)	12.81 (0.39)	12.81 (0.42)	17.50 (0.41)	17.49 (0.41)	17.41 (0.42)	17.47 (0.44)
Ratio P/N	1.19 (0.05)	1.14 (0.05)	1.11 (0.04)	1.15 (0.06)	0.70 (0.05)	0.68 (0.05)	0.71 (0.05)	0.70 (0.06)

### 3.2 Clone identification

A total of 94 leaves or bark samples (82 trees and 12 living roots on dead stumps) were successfully genotyped. Intraspecific variation within the study sites was examined with the 36‐SNP panel (Isabel et al., *submitted*). Three of the 36 SNPs were partially informative, and one was not informative because they did not vary among the 94 samples, then they were excluded (File S1). The clonal structure varied among the six sites (Table 4). The analysis showed that sites AB3 and QC3 were composed of five and three different clones, respectively (Table 4). The minimal number of allelic differences between two clones from the same site was 14 (QC3) and 17 (AB3). In the remaining sites and within each stand, all excavated stems belonged to the same clone, that is, with no allelic differences between each other (Table 4). All living roots from dead stumps had genotypes corresponding to at least one living tree in the site.

**Table 4:** Clones identification, natural root grafting between trees, and between trees and living roots from dead stumps in the six sites of balsam poplar in Alberta and Quebec

Sites	Alberta			Quebec		
	AB1	AB2	AB3	QC1	QC2	QC3
Number of clones (unique genetic profile)	1	1	5	1	1	3
Percentage of interclonal root grafts (%) (Number of grafts)	0 (0)	0 (0)	0 (0)	0 (0)	0 (0)	18 (2)
Percentage of intraclonal root grafts (%)/(Number of grafts)	100 (12)	100 (8)	100 (3)	100 (7)	100 (4)	82 (9)
Percentage of dead stumps uncovered with living roots connected to living trees by root grafts or parental roots (%)/ (Number of dead stumps)	25 (3)	16.6 (2)	8.3 (1)	25 (3)	16.6 (2)	8.3 (1)

### 3.3 Clonal integration

Parental root connections (Fig. 1C) were found between at least two living trees in all stands except in the oldest stand (AB3) where one parental root connection was only found between a living tree and a dead stump. There was a significant difference in the mean distance between trees connected to each other by parental root between Alberta and Quebec (*F*_1,6.96_ = 8.21, *p* < .02, Table 5). The largest root system network observed in an excavated area integrated five living trees in site AB1. In contrast, no parental root connection was found between living trees in site AB3, which had a mean distance between trees of 5.49 ± 2.27 m. In the remaining sites, the mean distance between trees was AB1 = 3.3 ± 1.6 m, AB2 = 3.7 ± 1.6 m in Alberta and QC1 = 6 ± 2.8 m, QC2 = 5.2 ± 2.2 m and QC3 = 3.5 ± 1.7 m in Quebec. There was a significant interaction between Province x Parental root connections between trees (*F*_1,545_ = 7.37, *p *<* *.006, Table 5) showing that the distance between trees connected by parental root was significantly lower than the distance between unconnected trees (0.47 ± 0.25 m vs. 3.14 ± 0.15 m and 1.55 ± 0.27 m vs. 4.25 ± 0.13 m) in Alberta and in Quebec, respectively. Mean distance between unconnected trees by parental roots was similar in Alberta and Quebec (3.14 ± 0.16 m vs. 4.25 ± 0.25 m). Overall, 22% and 25% of trees were connected with parental roots, respectively, in Alberta and in Quebec. There was no significant difference in the frequencies of trees connected by parental root between provinces (χ^2^ = 0.55, d.f. = 1, *p* = .4).

**Table 5:** Analyses of variance showing sources of variation, degrees of freedom (DF, DFD), and *F* and *Pr* values for distance between trees connected or not by parental roots and root grafts

Source of variation	DF	DFDen	*F*value	*Pr* values
Distance between trees connected or not by parental root				
Provinces (Alberta/Quebec)	1	6.96	8.21	**0.024**
Parental root connection (PRC)	1	545.2	80.58	**<0.001**
Provinces*PRC	1	545.2	7.37	**0.006**
Distance between trees connected or not by root grafts				
Provinces (Alberta/Quebec)	1	6.478	0.48	0.511
Root graft connection (RGC)	1	599.1	91.22	**<0.001**
Provinces*RGC	1	599.1	0.008	0.924

Bold indicates significant effects.

Trees were also connected by root grafts (Fig. 1D) in all excavated sites, and there was no significant difference in mean distance between trees connected by root grafts in Alberta and in Quebec (*p *=* *.51). Mean distance between trees or between trees and dead stumps with living roots connected by root grafts was significantly lower (*F*_1,599_ = 91.2, *p* < .001, Table 5) than the mean distance between trees which were not connected by root grafts (1.14 ± 0.17 m vs. 3.98 ± 0.11 m). Within the excavated sites, 53% and 48% of trees were linked with a root graft to at least one other living tree or to a dead stump with living roots in Alberta and Quebec, respectively. There was no significant difference in the frequencies of trees connected by root grafts across provinces (χ^2^ = 0.17, d.f. = 1, *p* = .6). Self‐root grafts (within the root system of a single tree) were found in the root system of every tree, but were not tallied. In site QC3, two trees of the most represented clone in the site (50% of trees belonged to this clone) formed root grafts with trees from different clones (representing 18% interclonal root grafts; Fig. 1D). No interclonal root grafts were found in the multiclonal site of Alberta (AB3). The age of root grafts ranged 7–47 years old. The excavation exposed a total of 12 dead stumps with living roots, which were connected to the roots of living trees, by parental roots or root grafts (Table 4). Lastly, 41% and 47% of excavated trees in Alberta and Quebec, respectively, were not connected to other trees within the excavated area by parental roots or root grafts.

## 4 Discussion

Despite abundant production of airborne seeds by balsam poplar trees (Bessey, 1904; Lee, Mahoney, & Rood, 1991; Walker, Zasada, & Chapin, 1986), all sampled trees in this study were from sucker origin, in contrast to subarctic Quebec where a number of balsam poplar stands were found to have originated from seeds (Comtois, Payette, & Simon, 1989). This result was unexpected, as although root suckering was a known reproduction mechanism for this species, it was thought to be of lesser importance than for aspen under nonarctic conditions (Peterson & Peterson, 1992). Our hypothesis that there would be more regeneration by seed in Quebec due to more precipitation is thus not verified, at least for these upland sites under boreal forest conditions. Perhaps, seed regeneration serves the species more for colonizing new areas, such as exposed mineral soil along road sides (Fortin, 2008; Laquerre, Leduc, & Harvey, 2009; Zasada et al., 1981), riparian areas (Rood et al., 1994), or in intensively burnt coniferous forests. Indeed, balsam poplar stands and associated understories do not readily support fire because of their higher moisture content, less flammable leaf chemical content, and the fact that they provide less fuel during the fire season (Keyser, Smith, & Shepperd, 2005; Moretti, Conedera, Duelli, & Edwards, 2002; Proença, Pereira, & Vicente, 2010). Consequently, unburned accumulated organic matter does not create favorable seedbeds for germination (Facelli & Pickett, 1991). Therefore, it seems that balsam poplar stands are more likely to regenerate by root sucking after fire while other forest types that burn more intensively may be invaded by seed regeneration. One could also argue that the preponderance of sucker regeneration found in this study is an indication of less intense fires and that greater fire intensity would have favored regeneration by seed. It is also possible that seed regeneration occurred alongside the sucker regeneration following the fire, but only those originating from root suckering remained in the six studied stands at the time of excavation some 43–103 years later. Extensive root suckering is however consistent with previous studies that reported abundant root suckering of the species in upland sites of Alaska following harvesting (Edwards & Dunwiddie, 1985; Zasada et al., 1981) and in areas relatively far away from riparian areas (Rood et al., 1994; Shaw, 1991). Reports of vegetative regeneration by root suckering by its close relative *Populus trichocarpa* are scarce but have been reported to occur (Roe, 1958).

The use of 36 SNP markers allowed us to confirm that four of the six studied stands were of clonal origin, as all trees within each site had the same clonal identity. The two other sites (AB3 and QC3) were also of clonal origin, but their relatively high level of genotypic diversity is an indication that seed regeneration must have occurred in the past and that the different genotypes were maintained and expanded vegetatively after stand‐replacing disturbances such as forest fires. Similar genotypic diversity has been found in aspen stands of eastern Canada (Namroud, Park, Tremblay, & Bergeron, 2005). In the later study, many unique genotypes were found (clones represented by single ramets), and unless the presence of parental roots was verified in the root system of these trees, it is impossible to certify that they were of seedling or sucker origin. We argue that the fact that trees can maintain root systems of dead trees by root grafting between different genotypes (Jelínková et al., 2009; this study) and that they are able to vegetatively regenerate these genotypes after a major disturbance can explain how clonal diversity is maintained over time in the absence of sexual reproduction. Our excavations uncovered many dead stumps with live roots, but these belonged to genotypes already present in the living trees. It does not however exclude the possibility of maintaining genotypes that have disappeared from the canopy and resprouting them after the next stand‐replacing disturbance, as root grafting was observed between roots of trees from different genotypes (Table 4).

This study provides further evidence that parental roots of trees regenerated from root suckering persist and integrate into the root system of the next generation of trees (DesRochers & Lieffers, 2001b). These results somewhat contradict earlier aspen literature which reported that either distal or proximal ends of the parental roots eventually rot away and make trees independent from each other (Gifford, 1966). The fact that these old parental roots can maintain roots of dead trees is also evidence that they remain functional over multiple disturbance events. Connected trees by parental root were significantly closer to one another in Alberta than in Quebec. We assume that after suckering, parental roots were enlarged in both acropetal and basipetal directions by the ramets which arose close to each other (Brown, 1935), creating parental roots with large diameters. The shorter mean distance between trees in Alberta could thus explain why parental roots on these sites were larger than the new roots on average, while they were slightly smaller at the Quebec sites where trees were further apart (Table 3). The relative longer mean distance between trees at the AB3 site (>5 m) as well as age‐related clonal fragmentation of clonal plants of arid and semiarid environments (Gatsuk, Smirnova, Vorontzova, Zaugolnova, & Zhukova, 1980) could also explain why no parental root connections between living trees were found at this site. However, trees were found connected to the roots of dead stumps, suggesting that natural self‐thinning in this site reduced the number of living connected trees by increasing distance between residual trees. We presume that large parental root connections have greater probability of dying, due to their large biomass and energy required to maintain them (DesRochers & Lieffers, 2001a). The obtained ratio P/N in Alberta indicates that parental roots were larger than the new roots as a whole, which is in accordance with DesRochers and Lieffers (2001b), who reported significant radial growth of parental roots following suckering in aspen from the same region. P/N ratios were smaller in Quebec and the difference could be related to the fact that size of parental roots at the time of suckering was smaller in Quebec compared to Alberta; less energy was drained into maintaining this large parental root network, and trees invested more energy into new roots in Quebec.

Trees were also connected by root grafts in all excavated sites (Table 4), indicating that root grafting is a common occurrence in balsam poplar, similar to what was found in aspen (DesRochers & Lieffers, 2001b; Jelínková et al., 2009), in *Pseudotsuga menziesii* (Eis, 1972), and in *Pinus banksiana* (Tarroux & DesRochers, 2010). It is generally accepted that root graft formation requires firm physical contact and pressure developed as roots increased in size, where intervening bark is broken down and vascular continuity is established (Bormann, 1966; Eis, 1972; Kozlowski & Cooley, 1961). Root grafting therefore may be facilitated between roots of adjacent trees compared with trees which were more distant from one another. Spatial distribution of trees also explained the frequency of root grafting incidence in other species, with more root grafting occurring between closely located trees (Reynolds & Bloomberg, 1982; Tarroux, DesRochers, & Krause, 2010). The percentages of grafted trees in this study (53% in Alberta and 48% in Quebec) were however somewhat lower than in declining aspen stands (60%) (DesRochers & Lieffers, 2001b) or in *Pinus strobus* stands (75%) (Bormann, 1966) reputed by their capacity to form many root grafts, but somewhat concurred with observations in aspen stands in Quebec that reported similar levels (48%) of root grafting between trees of the same versus different clones (Jelínková et al., 2009). These results however contrast with studies in northern prickly ash (*Xanthoxylum americanum* Mill.), for example, that reported that because of the diffusion of chemical inhibitors, roots from adjacent clones either stopped growth or turned away as soon as they approached roots of different genotypes making interclonal grafting virtually impossible (Reinartz & Popp, 1987). The small frequency of interclonal grafts found in this study can easily be explained by the low clonal diversity found in the excavated sites.

Root grafts were initiated relatively late in stand development, when trees were 28–41 years old. The fact that root grafts did not form earlier could be explained by little contact between roots of small trees (Tarroux & DesRochers, 2011; Tarroux et al., 2010) or by the heavy self‐thinning phase in sucker‐regenerated stands (Schier, 1982) making grafts disappear early if they had occurred between close ramets. Self‐thinning as stands mature could also explain the lack of parental connections between trees; As trees grow older and stands mature, natural thinning tends to make trees further apart, and when the parental roots are so large, the energy needed to support them may not be available by the reminder of the clone and portions of the parental root system (including connecting roots) lost with time. Finally, the frequencies of independent trees in this study were probably overestimated, because trees may have been connected to trees outside the excavated plots, with parental roots or grafts.

This study constitutes the first large‐scale excavations carried out in naturally established upland balsam poplar stands in eastern and western Canada. All excavated trees in this study were of sucker origin, despite differing moisture regimes, soil conditions, or stand characteristics of the two regions, making the results applicable at a large scale. Most trees were interconnected through root grafts and parental roots connections, except for the oldest site in Alberta where trees were further apart than in the other sites. Root connections were also found to support roots of dead trees. This finding that trees grow on communal root systems should be considered in silvicultural scenarios, rather than consider balsam poplar trees as distinct individuals.

## Funding Information

This study was funded by a doctoral scholarship Bourse en Mileu Pratique Innovation to K.E. Adonsou provided by Fonds de Recherche Nature et Technologie du Québec, Natural Sciences and Engineering Research Council of Canada (NSERC) and Norbord Industries Inc. Additional financial support was provided by Alberta‐Pacific Forest Industries Inc., Norbord Industries Inc. and NSERC through a collaborative research and development grant to A. DesRochers (Grant /Award number : CRDPJ‐381553‐09).

## Conflict of Interest

None declared.
